# Overexpression of Chickpea Defensin Gene Confers Tolerance to Water-Deficit Stress in *Arabidopsis thaliana*

**DOI:** 10.3389/fpls.2019.00290

**Published:** 2019-03-12

**Authors:** Manoj Kumar, Mohd Aslam Yusuf, Pooja Yadav, Shiv Narayan, Manoj Kumar

**Affiliations:** ^1^Department of Biosciences, Integral University, Lucknow, India; ^2^Department of Biotechnology, CSIR-National Botanical Research Institute, Lucknow, India; ^3^Department of Bioengineering, Integral University, Lucknow, India; ^4^Academy of Scientific and Innovative Research (AcSIR), Ghaziabad, India; ^5^Plant Physiology Laboratory, CSIR-National Botanical Research Institute, Lucknow, India

**Keywords:** *Arabidopsis thaliana*, *Ca-AFP*, defensin, water-deficit stress, mannitol, polyethylene glycol, transgenic

## Abstract

Plant defensins are mainly known for their antifungal activity. However, limited information is available regarding their function in abiotic stresses. In this study, a defensin gene, *Ca-AFP*, from *Cicer arietinum*, commonly known as chickpea, was cloned and transformed in *Arabidopsis thaliana* for its functional characterization under simulated water-deficit conditions. Under simulated water-deficit conditions (mannitol and polyethylene glycol-6000 induced), the transgenic *A. thaliana* plants had higher accumulation of the *Ca-AFP* transcript compared to that under non-stress condition and showed higher germination rate, root length, and biomass than the wild-type (WT) plants. To get further insights into the role of *Ca-AFP* in conferring tolerance to water-deficit stress, we determined various physiological parameters and found significant reduction in the transpiration rate and stomatal conductance whereas the net photosynthesis and water use efficiency was increased in the transgenic plants compared to that in the WT plants under water deficit conditions. The transgenic plants showed enhanced superoxide dismutase, ascorbate peroxidase, and catalase activities, had higher proline, chlorophyll, and relative water content, and exhibited reduced ion leakage and malondialdehyde content under water-deficit conditions. Overall, our results indicate that overexpression of *Ca-AFP* could be an efficient approach for conferring tolerance to water-deficit stress in plants.

## Introduction

Drought tolerance is the ability of plants to resist water-deficit conditions with low tissue water potential (Ingram and Bartels, 1996). A significant alteration in the expression of genes related to various physiological, regulatory, and molecular functions, which could be upregulated or downregulated, is reported in plants under stress conditions (Ingram and Bartels, 1996; Blum, 1998; Trethowan et al., 2001; Ramanjulu and Bartels, 2002; Bartels and Sunkar, 2005). Under water-deficit conditions in soil, plants adopt mechanisms either to escape or resist the stress (Levitt, 1980; Price et al., 2002). Traits like reduced epidermal (stomatal and cuticular) conductance, radiation absorption, and evaporative surface together with improved root architecture are some of the response of plants exposed to such conditions (Price et al., 2002).

The role of several plant proteins has been studied in transgenic *Arabidopsis thaliana* plants under osmotic stress; these include AtFP3 (Zhang et al., 2016), VvMBF1 (Yan et al., 2014), AtRabG3e (Mazel et al., 2004), EcGBF3 (Ramegowda et al., 2017), CarNAC4 (Yu et al., 2016), and GmDhn8 (Maitra and Cushman, 1998). Plant defensins are known for their important roles in biotic stress, especially against fungal pathogens. For example, *Pisum sativum* defensin 1 (Psd1) is involved in the response against *Neurospora crassa* (Lobo et al., 2007), *Medicago truncatula* defensin (MtDef4) is effective against *Fusarium graminearum* (Sagaram et al., 2013), and *Nicotiana alata* defensin (NaD1) has growth inhibitory activity against *Fusarium oxysporum* (Lay et al., 2012). Plant defensins have also been reported to play an important role during abiotic stresses. The soybean defensin gene (*Dhn8*) was demonstrated to be induced by drought (Maitra and Cushman, 1998) and *Nicotiana* defensins, *NeThio1* and *NeThio2*, were induced by NaCl-induced salinity stress (Komori et al., 1997). The pepper defensin gene *(CADEF1)* was shown to be induced by drought and salinity stresses in *Capsicum annuum* (Do et al., 2004). The expression of a plant defensin, *AhPDF1.1*, from zinc-hyperaccumulating *Arabidopsis halleri*, under the control of 35S promoter conferred Zn tolerance in transgenic *A. thaliana* plants (Mirouze et al., 2006).

Plant defensins are small, basic, cysteine-rich peptides, found ubiquitously in the plant kingdom (Osborn et al., 1995; Broekaert et al., 1997; Shewry and Lucas, 1997; Osborn and Broekaert, 1999; Thomma et al., 2002) that exhibit three-dimensional folding pattern stabilized by eight cysteine residues linked by four disulfide bridges (Broekaert et al., 1995; Almeida et al., 2002). More than 300 defensin-like genes have been identified in the model plant *A. thaliana* till date (Silverstein et al., 2005). These have been isolated from seeds (Broekaert et al., 1995; Thomma et al., 2003), roots (Sharma and Lönneborg, 1996), leaves (Segura et al., 1998; Do et al., 2004), and pods (Chiang and Hadwiger, 1991). We identified a defensin gene, *Ca-AFP*, along with several other drought-responsive differentially expressed genes, in our transcriptome sequencing of chickpea (*Cicer arietinum*) root samples exposed to water-deficit conditions (Table 1). The expression of these genes was validated through quantitative real time PCR (qRT-PCR) and *Ca-AFP* was observed to be upregulated under water-deficit conditions. In the present study, we investigated, for the first time, the role of chickpea defensin gene under water-deficit conditions by overexpressing it in *A. thaliana*. Our results show that *Ca-AFP* confers tolerance to water-deficit stress in transgenic *A. thaliana* plants and could, therefore, be used for generating drought-tolerant commercially important plants.

**Table 1:** Chickpea drought responsive genes identified through transcriptome analysis.

S.N.	LOC ID	log2(fold_change)	GeneID	Chromosome	Description of gene	Type_of_gene
1	LOC101512255	2.1274	101512255	Ca1	geraniol 8-hydroxylase-like	protein-coding
2	LOC101512255	–2.95378	101512255	Ca1	geraniol 8-hydroxylase-like	protein-coding
3	LOC101508810	–1.51518	101508810	Ca7	uncharacterized LOC101508810	protein-coding
4	LOC101508810	5.78519	101508810	Ca7	uncharacterized LOC101508810	protein-coding
5	LOC101503379	–2.22106	101503379	Ca3	RNA-binding protein 38-like	protein-coding
6	LOC101491913	–3.3973	101491913	Ca1	14 kDa proline-rich protein DC2.15-like	protein-coding
7	LOC101509326	1.54692	101509326	Ca6	proline-rich receptor-like protein kinase PERK2-like	protein-coding
8	LOC101513365	–2.59857	101513365	Ca4	GATA transcription factor 8-like	protein-coding
9	LOC101495554	–3.78102	101495554	Ca4	gibberellin-regulated protein 1-like	protein-coding
10	LOC101512021	5.91122	101512021	Ca1	defensin-like protein-like	protein-coding
11	LOC101490679	–2.0441	101490679	Ca8	abscisic acid 8′-hydroxylase 1-like	protein-coding
12	LOC101505927	–2.22819	101505927	Ca7	abscisic acid 8′-hydroxylase 1-like	protein-coding
13	LOC101496585	–3.16129	101496585	Ca7	36.4 kDa proline-rich protein-like	protein-coding
14	LOC101490393	–2.8389	101490393	Ca4	3-oxo-Delta(4,5)-steroid 5-beta-reductase-like	protein-coding
15	LOC101501606	–2.73243	101501606	Ca5	acidic mammalian chitinase-like	protein-coding
16	LOC101509704	–2.09366	101509704	Ca3	actin-3-like	protein-coding
17	LOC101494386	–3.46903	101494386	Ca6	aspartic proteinase PCS1-like	protein-coding
18	LOC101509714	–2.92187	101509714	Ca4	basic 7S globulin 2-like	protein-coding
19	LOC101495910	–1.75766	101495910	Ca1	basic 7S globulin-like	protein-coding
20	LOC101509822	–2.33358	101509822	Ca4	basic 7S globulin-like	protein-coding
21	LOC101510034	–2.25005	101510034	Ca4	basic 7S globulin-like	protein-coding
22	LOC101513089	–2.2806	101513089	Ca1	beta-fructofuranosidase, cell wall isozyme-like	protein-coding
23	LOC101513528	–1.56005	101513528	Ca1	beta-glucosidase 12-like	protein-coding
24	LOC101489749	2.08897	101489749	Ca5	bis(5′-adenosyl)-triphosphatase-like	protein-coding
25	LOC101496665	–3.26285	101496665	Ca5	brassinosteroid-regulated protein BRU1-like	protein-coding
26	LOC101494236	–1.90685	101494236	Ca8	calcium-transporting ATPase 2, plasma membrane-type-like	protein-coding
27	LOC101501496	–2.06753	101501496	Ca4	calmodulin-like protein 11-like	protein-coding
28	LOC101499686	1.72218	101499686	Ca5	cellulose synthase-like protein G1-like	protein-coding
29	LOC101496722	1.69591	101496722	Ca8	cinnamoyl-CoA reductase 1-like	protein-coding
30	LOC101509871	–1.87905	101509871	Ca7	COBRA-like protein 7-like	protein-coding
31	LOC101495399	–1.56719	101495399	Ca2	cysteine proteinase inhibitor-like	protein-coding
32	LOC101513755	–3.30691	101513755	Ca8	cytochrome P450 71D8-like	protein-coding
33	LOC101496712	1.73721	101496712	Ca2	cytochrome P450 83B1-like	protein-coding
34	LOC101491312	–1.7198	101491312	Ca8	cytochrome P450 84A1-like	protein-coding
35	XLOC_031917	–2.29121	101497136	Ca7	diacylglycerol kinase zeta-like	protein-coding
36	LOC101491646	–2.43042	101491646	Ca3	disease resistance response protein 206-like	protein-coding
37	LOC101504947	–2.0409	101504947	Ca6	disease resistance response protein 206-like	protein-coding
38	LOC101511741	–1.58078	101511741	Ca3	DNA ligase 1-like	protein-coding
39	LOC101490396	2.426	101490396	Ca5	eugenol synthase 1-like	protein-coding
40	LOC101514490	–1.99149	101514490	Ca6	expansin-like A2-like	protein-coding
41	LOC101489892	1.96486	101489892	Ca8	expansin-like B1-like	protein-coding
42	LOC101507544	–3.92787	101507544	Ca7	expansin-like B1-like	protein-coding
43	LOC101490554	–1.55537	101490554	Ca2	fasciclin-like arabinogalactan protein 2-like	protein-coding
44	LOC101500481	6.17303	101500481	Ca7	FBD-associated F-box protein At4g10400-like	protein-coding
45	LOC101505686	1.61881	101505686	Ca5	gamma-glutamyltranspeptidase 2-like	protein-coding
46	LOC101513567	–1.89271	101513567	Ca3	GDSL esterase/lipase CPRD49-like	protein-coding
47	LOC101514168	–2.91426	101514168	Ca6	geraniol 8-hydroxylase-like	protein-coding
48	LOC101514489	–3.41582	101514489	Ca6	geraniol 8-hydroxylase-like	protein-coding
49	LOC101489131	–1.52061	101489131	Ca1	glucan endo-1,3-beta-glucosidase-like	protein-coding
50	LOC101503489	–1.76305	101503489	Ca3	hevein-like preproprotein-like	protein-coding
51	LOC101504416	–2.14451	101504416	Ca7	histone H3.3-like	protein-coding
52	LOC101508417	–2.1537	101508417	Ca8	isoflavone reductase-like protein-like	protein-coding
53	LOC101498105	–3.17152	101498105	Ca7	L-ascorbate oxidase homolog	protein-coding
54	LOC101510707	–2.02723	101510707	Ca4	L-ascorbate oxidase homolog	protein-coding
55	LOC101511136	–2.28784	101511136	Ca5	L-ascorbate oxidase-like	protein-coding
56	LOC101495962	–1.7916	101495962	Ca3	L-gulono-1,4-lactone dehydrogenase-like	protein-coding
57	LOC101514307	–2.74197	101514307	Ca7	lysine-rich arabinogalactan protein 18-like	protein-coding
58	LOC101509335	–3.2507	101509335	Ca7	major latex allergen Hev b 5-like	protein-coding
59	LOC101500484	–1.95148	101500484	Ca2	mitochondrial uncoupling protein 5-like	protein-coding
60	LOC101503951	–1.54123	101503951	Ca4	MLP-like protein 34-like	protein-coding
61	LOC101500008	5.23079	101500008	Ca5	NAD(P)H-dependent 6’-deoxychalcone synthase-like	protein-coding
62	LOC101503208	2.51526	101503208	Ca5	NADH-ubiquinone oxidoreductase chain 5-like	protein-coding
63	LOC101510843	–2.29995	101510843	Ca7	NADP-dependent glyceraldehyde-3-phosphate dehydrogenase-like	protein-coding
64	LOC101491385	2.751	101491385	Ca6	non-cyanogenic beta-glucosidase-like	protein-coding
65	LOC101503860	1.7591	101503860	Ca5	non-specific lipid-transfer protein-like	protein-coding
66	LOC101511984	–2.31268	101511984	Ca4	patatin-2-Kuras 3-like	protein-coding
67	LOC101504619	–3.57814	101504619	Ca6	polygalacturonase inhibitor 2-like	protein-coding
68	LOC101506971	1.52448	101506971	Ca5	probable glutathione S-transferase parA-like	protein-coding
69	LOC101492282	1.8497	101492282	Ca3	probable non-specific lipid-transfer protein AKCS9-like	protein-coding
70	LOC101506095	–3.64235	101506095	Ca5	probable pectinesterase/pectinesterase inhibitor 25-like	protein-coding
71	LOC101489717	–2.13157	101489717	Ca3	probable pectinesterase/pectinesterase inhibitor 7-like	protein-coding
72	LOC101515495	1.62498	101515495	Ca7	probable peptide/nitrate transporter At3g54450-like	protein-coding
73	LOC101503329	–2.01431	101503329	Ca1	probable polygalacturonase-like	protein-coding
74	LOC101489781	–3.64932	101489781	Ca7	probable xyloglucan endotransglucosylase/hydrolase protein 23-like	protein-coding
75	LOC101493216	–2.41189	101493216	Ca5	probable xyloglucan glycosyltransferase 12-like	protein-coding
76	LOC101498412	1.50025	101498412	Ca5	protein EARLY FLOWERING 4-like	protein-coding
77	LOC101492463	–1.94625	101492463	Ca6	putative lipid-transfer protein DIR1-like	protein-coding
78	LOC101501102	–3.05622	101501102	Ca7	putative nuclease HARBI1-like	protein-coding
79	LOC101491061	–1.9557	101491061	Ca6	receptor-like protein kinase HERK 1-like	protein-coding
80	LOC101508898	–3.48617	101508898	Ca1	reticuline oxidase-like protein-like	protein-coding
81	LOC101490106	4.69002	101490106	Ca1	ribulose-phosphate 3-epimerase, cytoplasmic isoform-like	protein-coding
82	LOC101488811	2.10436	101488811	Ca1	serine carboxypeptidase-like 45-like	protein-coding
83	LOC101493761	2.52261	101493761	Ca6	serine hydroxymethyltransferase 1-like	protein-coding
84	LOC101513097	–3.47589	101513097	Ca7	snakin-2-like	protein-coding
85	LOC101503877	2.73941	101503877	Ca6	UDP-glucose flavonoid 3-O-glucosyltransferase 7-like	protein-coding
86	LOC101494020	1.55845	101494020	Ca2	uncharacterized LOC101494020	protein-coding
87	LOC101494177	–2.98733	101494177	Ca5	uncharacterized LOC101494177	protein-coding
88	LOC101494198	1.6535	101494198	Ca6	uncharacterized LOC101494198	pseudo
89	LOC101494609	–1.55765	101494609	Ca6	uncharacterized LOC101494609	pseudo
90	LOC101495255	4.69856	101495255	Ca6	uncharacterized LOC101495255	protein-coding
91	LOC101496044	7.03616	101496044	Ca7	uncharacterized LOC101496044	protein-coding
92	LOC101499217	–4.2123	101499217	Ca7	uncharacterized LOC101499217	protein-coding
93	LOC101500065	–1.89539	101500065	Ca8	uncharacterized LOC101500065	protein-coding
94	LOC101501038	–2.60722	101501038	Ca3	uncharacterized LOC101501038	protein-coding
95	LOC101508795	–1.99777	101508795	Ca6	uncharacterized LOC101508795	protein-coding
96	LOC101510669	2.23982	101510669	Ca8	uncharacterized LOC101510669	protein-coding
97	LOC101511932	–2.82384	101511932	Ca2	uncharacterized LOC101511932	protein-coding
98	LOC101512423	–3.25588	101512423	Ca5	uncharacterized LOC101512423	protein-coding
99	LOC101515113	1.63132	101515113	Ca4	uncharacterized LOC101515113	protein-coding
100	LOC101515418	–1.84621	101515418	Ca4	uncharacterized LOC101515418	protein-coding
101	LOC101489665	–4.03999	101489665	Ca1	xyloglucan endotransglucosylase/hydrolase protein 9-like	protein-coding

## Materials and Methods

### Plant Materials and RNA Isolation

Seeds of chickpea (*Cicer arientinum* L.) genotypes BG362 (drought tolerant) and P1003 (drought sensitive) were aseptically grown in Hoagland’s medium in a culture room at 24 ± 2°C under 16-h light: 8-h dark cycle, with a light intensity of ∼200 μmol m^-2^ s^-1^. Seven-day-old plants were subjected to polyethylene glycol (PEG 6000; SD Fine Chemicals Limited, India)-simulated osmotic stress for 4 days. The roots of PEG-treated and control samples were harvested and crushed in liquid N_2_. Total RNA was isolated using RNA Spectrum Plant Total RNA Kit (Sigma-Aldrich, United States).

*Arabidopsis thaliana* (Col-0 ecotype) seeds were surface-sterilized with Tween-20 for 5 min, and then with 70% ethanol for 5 min followed by 4% sodium hypochlorite (NaOCl; Sigma-Aldrich, United States) for 7 min, and were subsequently washed with autoclaved MilliQ water for 4–5 times. The sterilized seeds were stratified at 4°C for 3 days, sown in 300 g sterile soilrite filled in 10 cm × 10 cm (height × width) plastic pots, and kept at 22°C, under 75% relative atmospheric humidity and 16-h light:8-h dark cycle, with a light intensity of ∼200 μmol m^-2^ s^-1^ (Philips, Amsterdam, Netherlands).

### Validation, Isolation, and Sequence Analysis of *Ca-AFP* Gene

For validation of the expression of *Ca-AFP*, cDNA was prepared from stressed chickpea root and leaf samples from BG-362 and P-1003 genotypes and used for qRT-PCR analysis. cDNA was synthesized from 1 μg of total RNA using Verso cDNA synthesis kit (Thermo Fisher Scientific, United States). For qRT-PCR analysis, 10 μL reaction mixture contained 1 μL of cDNA, 1 μL each of forward and reverse primers (from 5 pmol stock), 5 μL SYBR green (Agilent Technologies, CA, United States), and 2 μL nuclease free water. The reaction was carried out in Stratagene Mx3000P (Agilent Technologies, CA, United States) using the following thermal cycling conditions: initial denaturation at 95°C for 2 min, followed by 40 cycles at 95°C for 15 s, 60°C for 20 s, and 72°C for 30 s. All the reactions were performed in triplicate. The fold-change in the expression of transcripts was calculated using the standard 2–ΔΔCT method (Livak and Schmittgen, 2001). The expression patterns of the transcripts were plotted in Microsoft Excel 2007.

The full length cDNA of *Ca-AFP* was isolated using RT-PCR from chickpea roots. The primer pair utilized for PCR amplification was as follows: forward primer: 5′-ATCAACAAATATATCAACCACACCA-3′ and reverse primer: 5′-TAATAATGAATATTTATTGTTGTTGTATATATG-3′. The *Ca-AFP* sequence was BLASTed using the NCBI online tool^1^. Multiple sequence alignment with other defensin proteins from *Cajanus cajan, M. truncatula, Medicago sativa, Phaseolus vulgaris, Tephrosia villosa, Vigna* radiata, and *Vigna angularis* was performed using Clustal Omega online tool^2^.

### Preparation of the Transformation Construct and Generation of Transgenic Plants

For overexpression of *Ca-AFP* in *A. thaliana*, the full-length cDNA was cloned under the control of *CaMV35S* promoter in pBI121 vector. For this, the full-length Ca-AFP gene was PCR-amplified from the cDNA prepared from the roots of chickpea BG-362 with gene-specific primers described above using PrimeSTAR GXL DNA Polymerase (Takara, Japan). The cDNA was cloned in an intermediate vector, pBluescript SK+ (Addgene Cambridge, MA, United States) by creating ends. The cloned cDNA was then excised from this vector by digestion with *BamH*I and *Eco53k*I and sub-cloned at the site for these restriction endonucleases in the binary vector, pBI121, in sense orientation. The resulting construct (*35S:Ca-AFP*) was introduced into *Agrobacterium tumefaciens* strain GV3101 using electroporation (Hercules, CA, United States). The transformation of *Arabidopsis* was done by *Agrobacterium*-mediated floral dip method (Clough and Bent, 1998). The seeds were harvested from the infiltrated plants and positive plants were selected on ½ Murashige and Skoog’s (MS) medium supplemented with 50 mg L^-1^ kanamycin (Sigma-Aldrich, United States). The kanamycin-resistant plants were transferred to soil after 8 days of germination and were grown in a growth chamber. *Arabidopsis* plants transformed with empty pBI121 vector (EV control) were also generated.

### PCR and qRT-PCR Analysis of Transgenic *A. thaliana* Plants

Genomic DNA (gDNA) was isolated from transgenic *Arabidopsis* lines using DNeasy mini prep kit (Qiagen, Germany). DNA quantification was done using NanoDrop spectrophotometer (Eppendorf, Germany). For PCR, the reaction mixture contained 100 ng gDNA, 2 μL 10× Taq buffer, 1 μL of 10 pmol each of forward and reverse gene-specific primers, 1 μL of 10 mM dNTP mixture (Genei Laboratories Pvt. Ltd., India), and 0.4 μL of Taq DNA polymerase (3 U/μL) (Genei Laboratories Pvt. Ltd., India), and the volume was made up to 20 μL using autoclaved MilliQ water. In the positive control reaction, 30 ng of the recombinant pBI121 plasmid was taken in place of gDNA. For negative control reaction, 100 ng gDNA of wild-type untransformed (WT) and EV transformed plants was taken. The PCR was performed under following conditions: initial denaturation at 95°C for 10 min followed by 30 cycles at 95°C for 45 s, 60°C for 30 s, and 72°C for 40 s, and a final extension at 72°C for 7 min. The PCR product was visualized by electrophoresis on a 1.2% agarose gel.

The expression of *Ca-AFP* in the transgenic plants was assessed using qRT-PCR. Total RNA was extracted from 100 mg leaves of the WT and transgenic lines using Spectrum Plant Total RNA Kit (Sigma Life Science, United States), according to manufacturer’s instructions. The quality and quantity of RNA samples were analyzed by agarose gel electrophoresis and NanoDrop spectrophotometer. The qRT-PCR was done taking three biological replicates using the above-mentioned primers and reaction conditions. *Actin2* of *A. thaliana* (*AtActin2*; GenBank accession number: U41998) was used as an internal control for normalization and was amplified using the primers: 5′-AGTAAGGTCACGTCCAGCAAGG-3′ (forward) and 5′-GCACCCTGTTCTTCTTACCGAG-3′ (reverse). The expression levels of genes (*ETHYLENE RESPONSE FACTOR1* and *VEGETATIVE STORAGE PROTEIN 1)* related to hormones like ethylene and jasmonic acid was also assayed through qRT-PCR. The sequences of primers designed for *A. thaliana AtERF1* (GenBank accession number: AT3G23240) were 5′-ACGTTCTCAACCGCCTACAG-3′ (forward) and 5′-CGGACTCGCTCTCTGGTG-3′ (reverse) and those designed for *AtVSP1* (GenBank accession number: AT5G24780) were 5′-TTTTACGCCAAAGGACTTGC-3′ (forward) and 5′-AATCCCGAGTTCCAAGAGGT3-3′ (reverse).

### Assessment of Water-Deficit Stress Tolerance of Transgenic *A. thaliana* Plants

The water-deficit stress tolerance of *Ca-AFP* overexpressing transgenic *A. thaliana* plants was analyzed using three homozygous lines (#1, #6, and #9). For this, the surface sterilized seeds of transgenic and WT *A. thaliana* were stratified at 4°C for 3 days and then inoculated in ½MS medium supplemented with mannitol (0, 100, 200, 250, and 300 mM) or PEG (0, 1.5, 3, 4.5, and 6%) and their germination rate was determined after 3, 6, and 9 days. Similarly, for root length and biomass measurement, 4-day-old seedlings of transgenic lines and WT were placed on ½MS medium containing different concentrations of mannitol (Sigma-Aldrich, United States) and PEG and the measurements were made after 10 days.

For assessment of the performance of transgenic lines and WT plants under simulated physiological drought conditions they were grown on soilrite in well-watered pots for 20 days. These plants were then subjected to water-deficit conditions by withholding water for the next 15 days. The control plants were watered regularly. The survival rate of plants was recorded. Thereafter, the plants were irrigated again for 5 days and their recovery was monitored. The leaf samples from plants exposed to well-watered and water-deficit conditions were harvested for different enzymatic assays. The total soluble protein was extracted from the leaf samples in bicarbonate buffer (Himedia, India) and quantified using the Bradford assay (Bradford, 1976) using Bradford reagent (Hercules, CA, United States). The previously described methods were used for the estimation of proline (Bates et al., 1973), superoxide dismutase (SOD) (Beauchamp and Fridovich, 1971), catalase (CAT) (Aebi, 1974), ascorbate peroxidase (APX) (Nakano and Asada, 1981), malondialdehyde (MDA), NADPH oxidase (NADPHox) (Cakmak and Marschner, 1988), peroxidase (POX) (Hemeda and Klein, 1990), and chlorophyll (Arnon, 1949). The accumulation of superoxide anion radical (O_2_^-^) and hydrogen peroxide (H_2_O_2_) in *Arabidopsis* plants transformed with *Ca-AFP* and empty vector (EV) and in WT plants was determined using nitrobluetetrazollium (NBT) and 3,3’-Diaminobenzidine (DAB) staining, respectively. The staining intensity of NBT and DAB in leaves was determined by densitometry using ImageJ software (ver. 1.46 for Windows 8) (Schneider et al., 2012). To measure the water loss, 3-week-old plants were exposed to 15 days of water-deficit condition as described above. The leaves from these plants were harvested and weighed immediately. These were placed on a dry filter paper at 25°C under an RH of 50–60% and weighed at designated time intervals (1, 2, and 3 h) after detachment. The water loss was calculated based on the initial fresh weight of plants. The experiment was conducted thrice for each transgenic and WT plant. To determine the electrolytic leakage (EL), leaves were rinsed with deionized water (dH_2_O) and immersed in 10 mL dH_2_0; they were then kept on a gyratory shaker at 100 rpm at room temperature. After 2 h, the conductivity (C1) of the samples was measured. Subsequently, the samples were boiled for 10 min and cooled to room temperature. The conductivities (C2) of the samples were measured again. The C1/C2 ratio was calculated to evaluate the relative electrolytic leakage from leaves samples (Liu et al., 2006).

The relative water content (RWC) was determined using rosette leaves of transgenic and WT plants exposed to water-deficit stress for different periods (3, 6, 9, 12, and 15 days). The detached leaves with intact petioles were immediately weighed for determining the leaf fresh weight (LFW) and then kept in falcon tubes containing 10 mL of dH_2_0. These leaves were kept for 5 h and allowed to imbibe water and were then weighed to obtain the leaf turgid weight (LTW). The leaves were then dried at 70°C for 5 h after wrapping in filter paper and weighed to obtain the leaf dry weight (LDW). The RWC was calculated using the formula: RWC = (LFW–LDW)/(LTW–LDW). The physiological parameters like transpiration rate, stomatal conductance, photosynthetic rate, and water use efficiency (WUE) were measured using Li-Cor 6800 gas exchange portable photosynthesis system (Li-Cor, Lincoln, NB, United States). The measurements were taken around 10:00 a.m. using three leaves from each plant. The stomatal conductance was used to determine the degree of stomatal opening and closing that defines the plant water status (Pei et al., 1997). For evaluation of stomatal size, epidermis of leaves of transgenic and WT plants were peeled and imaged under Leica DM 2500 microscope (Wetzlar, Germany) and the size measurements were made using the LAS V4.2 software (Lawson et al., 1998).

### Statistical Analysis

Statistical analysis was performed using the SPSS software (SPSS 16.0). Analysis of variance (ANOVA) was used to compare the significant differences (*p* < 0.05) based on Duncan Multiple Range Test (DMRT) in triplicate (*n* = 3).

## Results

### Validation, Characterization, and Cloning of the Ca-AFP Gene

**FIGURE 1:**
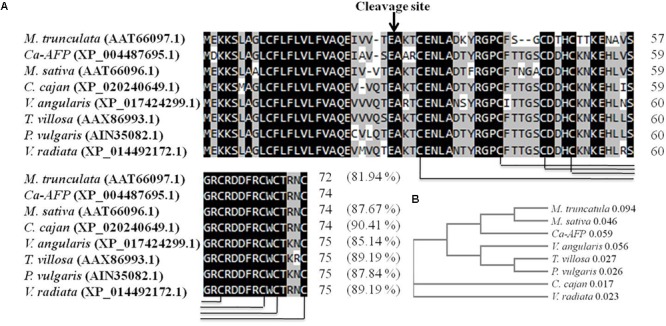
Comparison of *Ca-AFP* with other legume defensins **(A)** Multiple sequence alignment of *Ca-AFP* amino acid sequence with other legume defensin proteins using Clustal Omega software. **(B)** Phylogenetic analysis of *Ca-AFP* and other legume defensins. The defensin proteins whose sequences were used in this analysis were from *Cajanus cajan* (XP_020240649.1), *Medicago sativa* (AAT66096.1), *Tephrosia villosa* (AAX86993.1), *Vigna radiata* (XP_014492172.1), *Phaseolus vulgaris* (AIN35082.1), *Vigna angularis* (XP_017424299.1), and *Medicago truncatula* (AAT66097.1).

The Ca-AFP gene was identified to be overexpressed in chickpea plants exposed to water-deficit stress (Table 1). The validation of higher expression of *Ca-AFP* in the drought tolerant chickpea genotype was done using qRT-PCR analysis, the results of which showed its 4-fold upregulation in the roots and leaves of BG362 as compared to that in the drought sensitive (P-1003) genotype (Supplementary Figure S1). The Ca-AFP gene (LOC101512021) is located on chickpea chromosome number 1 (Ca1)^3^ and has a length of 542 bp and is predicted to encode a protein of 74 amino acids with a calculated MW of 8.3 kDa. The multiple sequence alignment of defensin proteins from different pulse crops showed that Ca-AFP shares 90.41, 87.67, 89.19, 89.19, 87.84, 85.14, and 81.94% similarity with the proteins from *C. cajan* (XP_020240649.1), *M. sativa* (AAT66096.1), *T. villosa* (AAX86993.1), *Vigna radiata* (XP_014492172.1), *P. vulgaris* (AIN35082.1), *V. angularis* (XP_017424299.1), and *M. truncatula* (AAT66097.1), respectively. All these proteins have eight common cysteine residues involved in four disulfide bridges (Figure 1A). The phylogenetic analysis revealed the identity of chickpea defensin with that of other pulses (Figure 1B). For overexpression of *Ca-AFP* in *A. thaliana*, the full-length cDNA was cloned into the plant expression vector pBI121 downstream of *CaMV35S* constitutive promoter (Supplementary Figure S2).

### PCR Screening and Expression of *Ca-AFP* in *A. thaliana*

A total of nine independent kanamycin resistant transgenic lines of *Arabidopsis* (T_1_ generation) were generated by floral dip method (Supplementary Figure S3A). Out of the nine lines, three homozygous transgenic lines (named as #1, #6, and #9) generated in the T3 generation were selected for further analysis (Supplementary Figure S3B). The PCR results showed the amplification of a 542-bp fragment of *Ca-AFP* confirming the integration of the transgene in the genome of all the nine *A. thaliana* lines (Supplementary Figure S3C). The expression of *Ca-AFP* in the transgenic *A. thaliana* leaves was assessed in the nine transgenic lines under control and water-deficit condition using qRT-PCR. The relative expression of *Ca-AFP* was found to be increased by 6.8-, 5.0-, 6.2-, 5.5-, 6.5-, 7.6-, 5.6-, 5.3-, and 7.0-folds in transgenic *Arabidopsis* lines #1, #2, #3, #4, #5, #6, #7, #8, and #9 under the water-deficit conditions compared to its expression in the respective plants under well-watered conditions (Supplementary Figure S3D).

### Response of Transgenic Plants to Mannitol and PEG Simulated Stress

**FIGURE 2:**
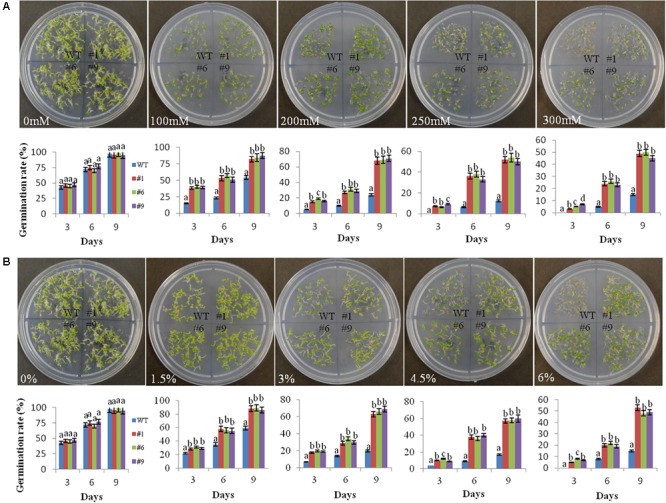
Germination rate of transgenic seeds under mannitol and polyethylene glycol (PEG) induced stress. Germination rates of seeds of the three transgenic lines (#1, #6, #9) and wild-type (WT) plants on MS medium supplemented with 100, 200, 250, and 300 mM mannitol **(A)** and on MS medium supplemented with 1.5, 3, 4.5, and 6% PEG-6000 **(B)** during a period from 3 to 9 days of stratification. At least 100 seeds were taken for each line in the plates and photographs were taken on the 9th day. The germination rates of seeds are shown graphically below each plate. Values are means ± SE (*n* = 3). Different letters above the bars indicate significant differences (*p* < 0.05) as analyzed by Duncan Multiple Range Test applied to different transgenic and WT lines.

The germination of seeds was normal under the control conditions in the WT as well as in the transgenic lines. However, the germination rate decreased in the WT compared to that in the transgenic lines under stress conditions. On 9th day of the experiment, the average germination rate of transgenic seedlings was 84, 69, 52, and 48% in comparison to 54, 24, 12, and 15% for WT in the presence of 100, 200, 250, and 300 mM mannitol, respectively (Figure 2A). The germination rates of the transgenic lines were significantly higher compared to that of WT under PEG simulated osmotic stress conditions. After 9th day, the average germination rates for the transgenic lines were 88, 66, 58, and 50% in comparison to 59, 20, 17, and 15% for WT in the presence of 1.5, 3, 4.5, and 6% PEG, respectively (Figure 2B).

**FIGURE 3:**
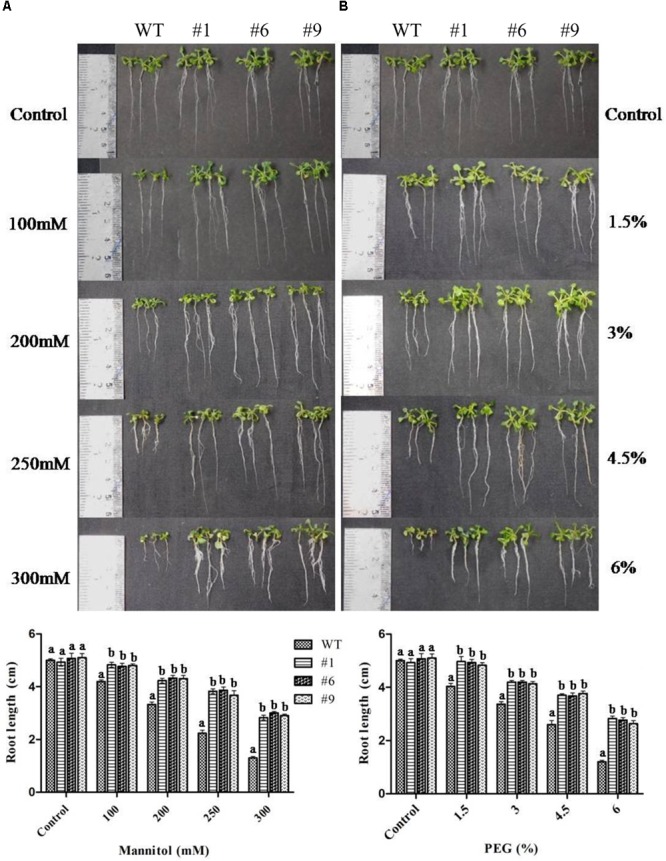
Root growth of transgenic plants under mannitol and polyethylene glycol (PEG) induced stress. Four-day-old seedlings of transgenic lines (#1, #6, #9) and wild type (WT) were grown on ½MS medium supplemented with 100, 200, 250, and 300 mM mannitol **(A)** or ½MS medium supplemented with 1.5, 3, 4.5, and 6% PEG **(B)**. Photographs were taken after 10 days. Values are means ± SE (*n* = 3). Different letters above the bars indicate significant differences (*p* < 0.05) as analyzed by Duncan Multiple Range Test applied to different transgenic and WT lines.

**FIGURE 4:**
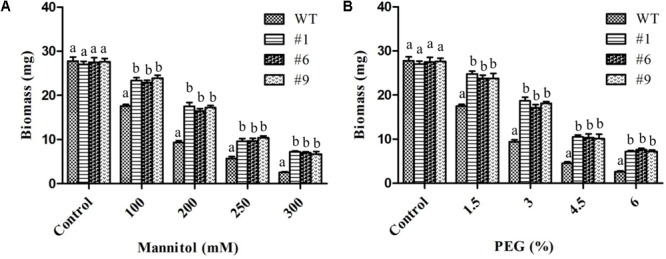
Biomass of transgenic plants under mannitol and polyethylene glycol (PEG) induced stress. *In vitro* grown transgenic plants were used for biomass measurements after 10 days of stress. Histograms showing biomass of plants exposed to 100, 200, 250, and 300 mM mannitol **(A)** or 1.5, 3, 4.5, and 6% PEG **(B)**. Values are means ± SE (*n* = 3). Different letters above the bars indicate significant differences (*p* < 0.05) as analyzed by Duncan Multiple Range Test applied to different transgenic and WT lines.

Overall, the root growth was reduced in the presence of mannitol and PEG. However, in comparison to WT, transgenic lines showed significantly longer roots. The average root lengths of the transgenic lines were 4.8, 4.3, 3.8, and 2.9 cm compared to 4.2, 3.3, 2.2, and 1.3 cm for WT in the presence of 100, 200, 250, and 300 mM of mannitol, respectively (Figure 3A). Similarly, the average root lengths of the transgenic lines were 4.9, 4.18, 3.7, 2.74 cm compared to 4, 3.4, 2.6, and 1.2 cm for WT in the presence of 1.5, 3, 4.5, and 6% PEG, respectively (Figure 3B). The average biomass of the transgenic lines was 1.3-, 1.8-, 1.7-, and 2.8-times higher compared to that of the WT plants in the presence of 100, 200, 250, and 300 mM mannitol, respectively; it was 1.4-, 1.9-, 2.3-, and 2.8-times higher than that of the WT plants in the presence of 1.5, 3, 4.5, and 6% PEG, respectively (Figure 4A,B).

### Tolerance of Transgenic Plants to Water-Deficit Conditions

**FIGURE 5:**
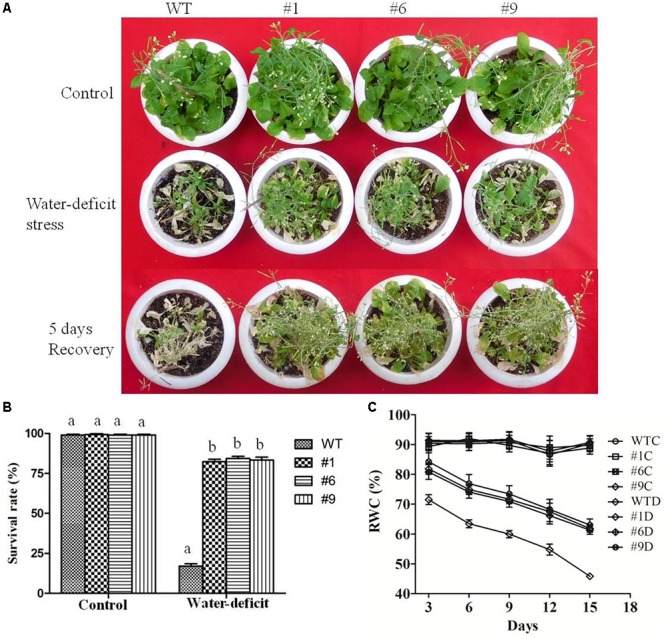
Water-deficit stress tolerance of *Arabidopsis thaliana* plants overexpressing *Ca-AFP.* **(A)** Transgenic *A. thaliana* (#1, #6, #9) plants under control and water-deficit (15 days) conditions. **(B)** Survival rates of the transgenic plants under water-deficit conditions. **(C)** Relative water content (RWC) of the transgenic plants. Values are means ± SE (*n* = 3). Different letters above the bars indicate significant differences (*p* < 0.05) as analyzed by Duncan Multiple Range Test applied to the transgenic and wild type (WT) lines.

**FIGURE 6:**
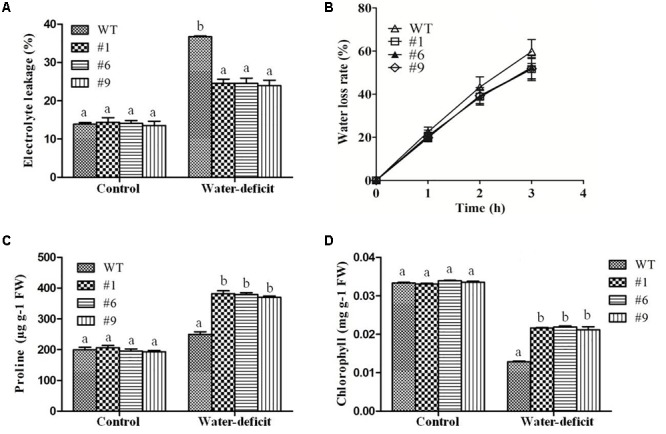
Physiological changes in transgenic plants under water-deficit condition. Electrolytic leakage (EL) **(A)**, rate of water loss **(B)**, proline content **(C)**, and chlorophyll content **(D)** in transgenic (#1, #6, #9) lines and wild-type (WT) plants. Values are means ± SE (*n* = 3). Different letters above the bars indicate significant differences (*p* < 0.05) as analyzed by Duncan Multiple Range Test applied to the transgenic and WT lines.

To assess the tolerance of *Ca-AFP* overexpressing transgenic *A. thaliana* plants to water-deficit stress, they were grown in soilrite for 3 weeks in a growth chamber and were then exposed to water-deficit stress by withholding of water for the next 15 days (Figure 5A). Under well-watered conditions, the growth of the transgenic and WT plants was identical. However, under water-deficit condition only 17% of the WT plants survived whereas about 84% survival rate was recorded for the transgenic plants (Figure 5B). After 5 days of recovery, most of the transgenic plants recovered but very low recovery was observed for the WT plants. We also measured the biomass and root length in all the nine lines of transgenic *Arabidopsis* plants, and EV transformed and WT plants exposed to 15 days of water-deficit conditions. Under well-watered conditions, transgenic plants and control plants showed almost similar biomass. However, biomass and root length were upto 2- and 1.8-fold, respectively, in the transgenic plants compared to that in the WT and EV transformed plants under water-deficit conditions (Supplementary Figure S4). The biomass of EV transformed and WT plants was almost the same under water-deficit conditions. Moreover, transgenic, WT, and EV transformed plants were evaluated for tolerance to water-deficit stress imposed for 15 days at pre-bolting stage and subsequent recovery for 5 days. In this case, the WT and EV transformed plants died whereas more than 85% transgenic plants survived (Supplementary Figure S5). The water loss and antioxidant parameters were measured after 15 days of imposition of water-deficit condition whereas RWC was measured after 3, 6, 9, 12, and 15 days. The values of average RWC for the transgenic plants were 82, 75, 72, 67, and 62% whereas the same for WT were 71, 63, 60, 54, and 45% after 3, 6, 9, 12, and 15 days of water-deficit treatment. The average RWC of the transgenic plants was 62% whereas it was 42% for the WT plants (Figure 5C). The average EL in the transgenic lines was 24.35% compared to 37% in the WT plants (Figure 6A) whereas the average water loss after 1, 2, and 3 h was 20, 39, and 52% in the transgenic plants compared to 22.5, 43, and 60% in the WT plants under water-deficit condition (Figure 6B). The contents of proline and chlorophyll were found to be increased in the transgenic plants. In the transgenic lines #1, #6, and #9, the proline content was determined to be 1.55-, 1.5-, and 1.5-fold higher, respectively, than the content in the WT plants under the water-deficit condition (Figure 6C). The chlorophyll content was found to be 1.7-, 1.7-, and 1.65-fold higher in the transgenic lines #1, #6, and #9, respectively, compared to that in the WT plants under water-deficit conditions (Figure 6D).

**FIGURE 7:**
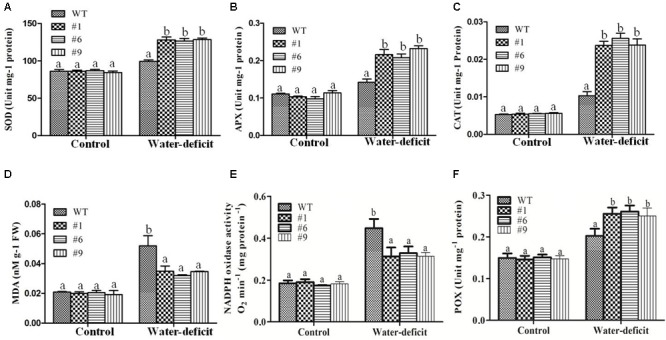
Changes in activities of superoxide dismutase (SOD), ascorbate peroxidase (APX), catalase (CAT), malondialdehyde (MDA), NADPH oxidase (NADPHox), and apoplastic peroxidise (POX) content under water-deficit condition. Activities of SOD **(A)**, APX **(B)**, CAT**(C)**, MDA **(D)**, NADPHox **(E)** and POX **(F)** content in the wild-type (WT) and transgenic *A. thaliana* (#1, #6, and #9) lines under control and water-deficit conditions. Values are means ± SE (*n* = 3). Different letters above the bars indicate significant differences (*p* < 0.05) as analyzed by Duncan Multiple Range Test applied to the different transgenic and WT lines.

The results of assays for SOD, APX, CAT, NADPHox, POX, and MDA did not show any significant differences between the transgenic lines and WT plants under normal conditions whereas under water-deficit condition, the average SOD, APX, and CAT activities were 1.3-, 1.50-, and 2.40-fold higher in the transgenic plants compared to that in the WT plants, respectively (Figure 7A–C). The MDA content was lower in the transgenic plants compared to that in the WT plants under water-deficit stress (Figure 7D). The NADPHox activity was found to be significantly decreased in transgenic plants compared to that in WT plants whereas the activity of apoplastic POX was found to be significantly increased in transgenic plants compared to that in WT plants under water-deficit condition (Figure 7E,F). A comparative assessment of the accumulation of two major ROS, O_2_^-^ and H_2_O_2_, in the different lines was done by histochemical staining. Under non-stressed control condition no staining was observed in any of the lines. However, after 15 days of imposition of water-deficit stress, the WT and EV transformed plants showed dark blue (for NBT) and deep brown (for DAB) staining whereas the staining was much less in the *Ca-AFP* transformed lines, suggesting lower accumulation of these ROS in the *Ca-AFP* transformed lines (Supplementary Figures S6A,B). A comparative analysis of the staining intensities is presented in Supplementary Figures S6C,D. The fact that the EV transformed plants behaved similar to the WT plants with respect to their accumulation of ROS as well as biomass and root length under control and water-deficit stress conditions suggests that the better performance of the *Ca-AFP* transformed lines vis-à-vis the WT and EV plants was related to the expression of *Ca-AFP* in these lines.

**FIGURE 8:**
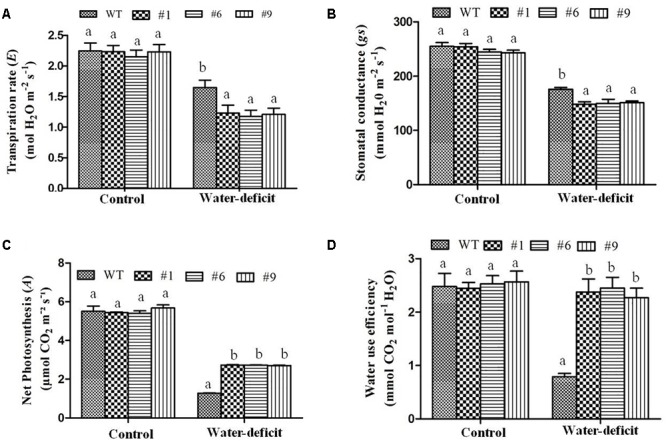
Physiological parameters of transgenic plants under water-deficit condition. **(A)** Transpiration rate, **(B)** stomatal conductance, **(C)** photosynthetic rate, and **(D)** water use efficiency in the wild-type (WT) and transgenic *A. thaliana* (#1, #6, and #9) lines under control and water-deficit conditions. Values are means ± SE (*n* = 3). Different letters above the bars indicate significant differences (*p* < 0.05) as analyzed by Duncan Multiple Range Test applied to different transgenic and WT lines.

In the WT plants, the MDA content was found to be 1.53-fold higher than in the transgenic plants. The transpiration rate and stomatal conductance were measured under well-watered and water-deficit stress conditions in the WT and transgenic plants. The stomatal closure, which enhances the water-deficit tolerance, was observed in the transgenic plants. The transpiration rate and stomatal conductance were found to be higher in the WT compared to those in the transgenic plants under water-deficit stress (Figure 8A,B). The average transpiration rate and stomatal conductance in the transgenic plants were 1.2 mol H_2_O m^-2^ s^-1^ and 150 mmol H_2_O m^-2^ s^-1^ compared to 1.64 mol H_2_O m^-2^ s^-1^ and 176 mmol H_2_O m^-2^ s^-1^ in the WT plants, respectively. The photosynthetic rate was found to be normal in the WT and transgenic plants under well-watered conditions whereas it decreased in both under the water-deficit conditions. The photosynthetic rate was 2.7-fold and WUE was 3-fold higher in the transgenic plants than in the WT plants under water-deficit conditions (Figure 8C,D). The size of guard cells (width and length) in the leaf epidermis of WT plants was significantly larger compared to that in the transgenic lines (Supplementary Figure S7). The average guard cell width to length dimension was 14.7 by 25.5 μm for the WT, in contrast to 12.8 by 21.5 μm, 13.2 by 22.5 μm, and 12.3 by 22 μm for transgenic lines (#1, #6, #9). Therefore, *Ca-AFP* expression decreased the stomatal size in transgenic *Arabidopsis* lines resulting in the reduction of water loss rates and transpiration efficiency that probably conferred tolerance to water-deficit stress.

Methyl jasmonate plays an important role in plant defense and in various developmental pathways including seed germination, root growth, flowering, fruit ripening, and senescence. ABA plays highly diverse roles in plants and it has been well known to promote leaf senescence. Therefore, we analyzed the effects of ABA and methyl jasmonate on germination rate and leaf senescence of transgenic and WT plants. Leaf disks of WT plants were damaged after 16 h of incubation in 5 μM ABA and 5 μM MeJA containing medium under dark condition (Supplementary Figure S8A). However, leaf disks of transgenic plants were less deteriorated compared to those of WT. Surface sterilized seeds of transgenic and WT were inoculated on ½ MS medium containing 5 μM ABA or 5 μM MeJA and then the germination rate was measured after 4 days of inoculation. The average germination rate of WT and transgenic seeds were 79 and 78% on ½MS medium (control). The average germination rates of transgenic lines were 53 and 58% in 5 μM ABA and 5 μM MeJA, respectively (Supplementary Figures S8B,D), whereas germination rates of WT seeds were 21 and 20% in 5 μM ABA and 5 μM MeJA, respectively. The root length of germinated seedlings of transgenic lines was higher than that of WT under ABA and MeJA treatments (Supplementary Figure S8C). The expression levels of both the hormone-related genes, *AtERF1* and *AtVSP1*, were elevated under water-deficit condition in transgenic lines as well as in WT compared to the levels in well-watered plants. It was observed that in transgenic and WT plants, the expression of *AtERF1* was upregulated 6- and 2.5-fold, respectively, under water-deficit condition. Similarly, the expression of *AtVSP1* was upregulated 8-fold in transgenic lines and 3-fold in WT plants (Supplementary Figures S9A,B).

## Discussion

Chickpea is one of the most important pulse crops cultivated worldwide and forms a rich source of dietary protein. Studies over the past decade have revealed a huge amount of information related to chickpea genome (Jain et al., 2013; Varshney et al., 2013; Gupta et al., 2016) and transcriptome analyses of this plant under stress conditions have been reported (Hiremath et al., 2011; Agarwal et al., 2012; Jhanwar et al., 2012; Singh et al., 2013; Pradhan et al., 2014; Garg et al., 2016; Iquebal et al., 2017). Herein, we report the cloning and characterization of *Ca-AFP* and its *Agrobacterium*-mediated genetic transformation in *A. thaliana*. The Ca-AFP gene encodes an 8.3-kDa protein containing 78 amino acids. Plant defensins display a conserved tertiary structure that is made up of a triple-stranded antiparallel β-sheet and one α-helix, stabilized into a compact shape by the disulfide bridges. These disulfide bridges develop a cysteine-stabilized α-helix β-sheet motif (CSα/β) (Kobayashi et al., 1991; Zhu et al., 2005). Apart from CSα/β motif, two additional conserved motifs, named α-core, surrounding the loop linking the first β-strand to the α-helix, and the γ-core containing the hairpin loop that connect β-strands 2 and 3 (Lβ2β3) were also found in the defensin structure (Yount and Yeaman, 2004; Yount et al., 2007). *Ca-AFP* has a typical signal peptide similar to all the other reported plant defensins (Meyer et al., 1996). Moreover, *Ca-AFP* is significantly identical to XP_020240649.1, AAT66096.1, AAX86993.1, XP_014492172.1, AIN35082.1, XP_017424299.1, and AAT66097.1, because of which these proteins lie in the same subgroup in the phylogenetic analysis (Figure 1A,B). *Triticum aestivum* defensin 1 (TAD_1_) gene showed tolerance to cold stress in *T. aestivum* (Koike et al., 2002). Under the control of the CAMV35S promoter, *A. halleri* defensin gene conferred Zn tolerance in *A. thaliana* plants (Mirouze et al., 2006). Although defensins are mainly known to be upregulated in response to biotic stress, their induction under abiotic stress, such as drought, has been observed (Govind et al., 2009). We used the CaMV35S promoter for over-expression of *Ca-AFP* in *A. thaliana* for improving water-stress tolerance. The qRT-PCR analysis of the generated transgenic lines showed an upregulation of the *Ca-AFP* gene under water-deficit condition (Supplementary Figure S3D). To characterize the mechanisms involved, mannitol and PEG 6000 were used to simulate water deficit conditions. Three homozygous (T_3_) transgenic lines of *A. thaliana* were used for the stress tolerance assay. These transgenic *Arabidopsis* plants showed higher germination rate (Figure 2) under mannitol- and PEG-induced stress. The process of maintaining water relations under osmotic stress is termed as osmotic adjustment. The accumulation of solutes under water deficit conditions results in a decrease in the osmotic potential of the cell, which pulls water molecules inside the cells and helps in maintaining turgor. For artificial induction of osmotic stress, PEG (Rauf et al., 2007; Khakwani et al., 2011) and mannitol (Bohnert et al., 1999) are used widely and these solutes decrease the osmotic potential. Previous studies have shown that transgenic *Arabidopsis* plants overexpressing stress related proteins exhibit higher germination rate, root length, and biomass under mannitol and NaCl stress (Huang et al., 2015; Xu et al., 2015; Yu et al., 2016). In the present study, the root length in the transgenic lines was about 2-times more than in the WT in the presence of 300 mM mannitol and 6% PEG (Figure 3). Similarly, the transgenic lines also showed higher biomass compared to the WT plants under the induced stress conditions.

In earlier studies, transgenic *Arabidopsis* plants overexpressing stress related proteins showed higher survival rates compared to the WT plants under water deficit conditions (Yan et al., 2014; Huang et al., 2015; Yao et al., 2017). It was reported that the overexpression of *JcPR-10a*, an antifungal protein of *Jatropha curcas* caused reduction in the transpiration efficiency and increased WUE during biotic and abiotic stress in transgenic *Nicotiana tabacum* (Agarwal et al., 2016). It was also reported that a *C. annuum* gene, *CaPMEI1*, which encodes an antifungal pectin methylesterase inhibitor protein, enhanced disease resistance, as well as drought and oxidative stress tolerance in transgenic *Arabidopsis* plants. The *CaPMEI1* overexpressing *Arabidopsis* showed reduced transpiration and enhanced root elongation (An et al., 2008). In another study, an antimicrobial protein gene, *CaAMP1*, was found to be strongly induced in pepper leaves exposed to ABA, NaCl, drought, or low temperature. The overexpression of *CaAMP1* conferred enhanced tolerance to high salinity and drought in transgenic *Arabidopsis* plants. These transgenic plants were also highly tolerant to osmotic stress caused by higher concentrations of mannitol. The transgenic *Arabidopsis* plants overexpressing *CaAMP1* had stimulated growth of roots and decreased transpiration rate and enhanced stomatal closure that prevented water loss resulting in the protection of the plants from various environmental stresses (Lee and Hwang, 2009). The constitutive overexpression of a rose expansin gene in *A. thaliana* plants resulted in drought tolerance with decrease in water loss rates and higher RWC in the transgenic plants (Lu et al., 2013). A significant reduction in RWC of transgenic *A. thaliana* and WT plants was observed under drought stress (Brini et al., 2007) with RWC being upto 82% in the transgenic plants compared to 55% in the WT plants under drought conditions (Butt et al., 2017). Similarly, increased RWC was observed in the transgenic plants compared to that in the WT plants in the present study (Figure 5C). Furthermore, EL and MDA content were found to be normal in both the transgenic and WT whereas under water-deficit condition higher values were obtained in the WT plants compared to that in the transgenic plants (Figure 6A, 7D). Malondialdehyde is usually used as a marker for lipid peroxidation (Dong et al., 2003). After exposure of plants to drought/oxidative stress, the higher content of ROS generated in plants can be evaluated in terms of the accumulation of MDA (Tu et al., 2016). The rate of water loss was reported to be higher in the WT than in the transgenic *A. thaliana* plants expressing different stress-related proteins under drought stress (Brini et al., 2007; Yan et al., 2014). We also found higher rate of water loss in the WT plants compared to that in the transgenic plants (Figure 6B). The reduced rate of water loss in the transgenic plants resulted in water-stress tolerance.

The accumulation of osmoprotectants in plants has also been reported under stress conditions. Osmotic potential is a typical indicator of the osmotic adjustment ability under varying physiological conditions. It has been used as an efficient index to assess genotypes for tolerance against osmotic stress (Zhu, 2002). Proline is an important amino acid whose accumulation in plants indicates stress tolerance and it adjusts the intracellular osmoticum under drought conditions. It scavengers free radicals (Ashraf and Foolad, 2007), and is involved in the overexpression of genes responsible for drought tolerance (Sharma et al., 2011) and in protecting plant cells from damage (Kishor et al., 1995). We observed the accumulation of higher amounts of proline in transgenic plants under water-deficit condition (Figure 6C). Similarly, the chlorophyll content was found to be higher in the transgenic plants compared to that in the WT plants under water-deficit conditions (Figure 6D). The chlorophyll content correlated with higher net photosynthesis and confirmed that the transgenic plants could maintain better photosynthesis under drought stress (Figure 8C). The *C. annuum* defensin 1 gene was reported to be expressed in leaves under drought, salinity, and wound stress. The expression of this gene was not found under normal conditions (Do et al., 2004) and it was observed that plant defensins are involved in the adaptation of plants to environmental stresses.

The production of reactive oxygen species (ROS), and hence oxidative stress, most often plays a role in defensin-mediated cell death, as has been reported for many plant defensins, including RsAFP2 (Aerts et al., 2007), HsAFP1 (Aerts et al., 2011), DmAMP1 (Aerts et al., 2006), and NaD1 (van der Weerden et al., 2008). Plants can regulate the levels of ROS through antioxidant enzymes, such as SOD, APX, CAT and POX, which scavenge the ROS molecules to impart enhanced resistance to drought (Hameed et al., 2012; Cai et al., 2015; Xu et al., 2015; Kumar et al., 2016; Yao et al., 2017). The NADPHox catalyzes the production of superoxides, an important ROS in cells. These enzymes are conserved in plants, fungi and animals and are actively involved in ROS production (Torres and Dangl, 2005). In present study, the activity of NADPHox was found to be lower in transgenic lines under water-deficit conditions. However, no significant changes were seen under well-watered conditions. It reflects that the ROS production was lower in transgenic lines compared to that in WT plants correlating with the results of ROS assay in transgenic lines under water-deficit conditions. The accumulation of ROS is observed under abiotic stress conditions, like drought, that results in oxidative damage and cell death in plants. To reduce the excessive ROS accumulation, plants have developed a complex set of antioxidant strategies to eliminate their damaging effects and maintain redox homeostasis (Gill and Tuteja, 2010). Plants can regulate the ROS levels through modulation of ROS scavenging enzymes, such as SOD, APX, CAT, and MDA. SOD is the front-line enzyme in protection against ROS attack because it rapidly scavenges superoxide, one of the first ROS to be produced, dismutating it to oxygen and H_2_O_2_ (Bowler et al., 1992). The major enzymatic cellular scavengers of H_2_O_2_ are CAT and APX (Noctor and Foyer, 1998). APX, an enzyme located in every cellular compartment that produces ROS, might function as a fine regulator of steady-state levels of intracellular ROS, possibly for signaling purposes, whereas CAT located exclusively in the peroxisomes, might function as a bulk remover of excess ROS produced under stress conditions (Mittler, 2002). Our results showed that the SOD, APX, CAT, and POX activities were significantly higher in the *Ca-AFP* overexpressing transgenic plants under water-deficit condition (Figure 7A–C,F). Our study with the transgenic plants overexpressing defensin shows that better ROS homeostasis is involved in the tolerance of these plants to simulated drought stress.

**FIGURE 9:**
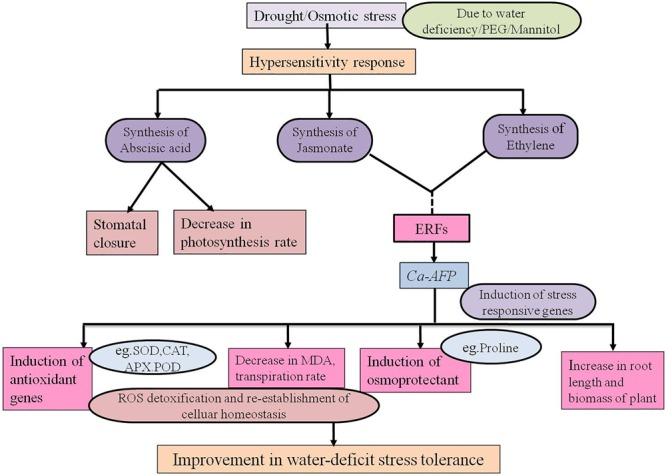
Proposed model for the regulatory network of the *Ca-AFP* response under water-deficit condition.

The regulation of stomata plays an important role in the exchange of gases between plants and atmosphere. The stomatal opening is responsible for 90% water loss from plants through transpiration (Wang et al., 2009). The reduction in stomatal conductance results in a decrease in net photosynthetic and transpiration rates with progressive water stress. It leads to increased WUE because transpiration is repressed more than photosynthesis (Xu et al., 2010). We observed that the transpiration rate and stomatal conductance were normal under well-watered conditions whereas under water-deficit conditions, the rates were significantly decreased in the transgenic plants (Figure 8A,B). The stomatal behavior could also be regulated by H_2_O_2_ for optimizing the WUE. The tolerance to drought was positively correlated with the increasing WUE in rice plants (Huang et al., 2009). Our results showed that the WUE was significantly increased in the transgenic plants under water-deficit conditions (Figure 8D). In a previous study, TAD1 was robustly and rapidly induced when wheat plants were exposed to cold stress whereas its expression was not induced by the plant hormones, such as ABA, salicylic acid, and methyl jasmonate (Brown et al., 2003; Do et al., 2004), that normally drive the expression of defensins. Its strong induction during exposure to cold implies that this plant defensin is involved in the adaptation of wheat to the cold. Production of plant defensins is also induced in response to environmental stress, such as drought (Maitra and Cushman, 1998), and signaling molecules, including methyl jasmonate, ethylene, and salicylic acid (Hanks et al., 2005). A hypothesized model for the role of defensin gene during water-deficit condition is proposed. A common regulatory defense mechanism explains the involvement of some antimicrobial peptides (AMPs). These peptides are generally induced against biotic stressors, especially defensin is induced by fungal pathogens. In addition to biotic stress, many of the abiotic stressors, for example, drought, wounding, cold, and salinity may induce the synthesis of defensins (Lay and Anderson, 2005). Ethylene Response Factor1 (ERF1) is known as an upstream component in both jasmonate (JA) and ethylene (ET) signaling and is involved in pathogen resistance. It was also reported that ERF1 was highly induced by high salinity and drought stress in *A. thaliana* (Cheng et al., 2013). ERF1 plays a positive role in salt, drought, and heat stress tolerance by stress-specific gene regulation, which integrates JA, ET, and abscisic acid signals. In response to various stress signals ERF1 binds to different *cis* elements (DRE element or GCC box). The induction of ERF1 required ET and JA signaling under abiotic stress and it was negatively regulated by ABA (Cheng et al., 2013). JA signaling enhances the activities of antioxidant enzymes, such as SOD, POD, CAT, and APX, as well as the tolerance to salinity stress in wheat (Qiu et al., 2014). ET causes closing of stomata in *Arabidopsis* through ethylene-induced H_2_O_2_ synthesis (Desikan et al., 2006). MeJA-mediated stomatal closure has been linked to cytoplasmic alkalinization in guard cells, production of ROS and NO, and activation of K^+^ efflux channels (Evans, 2003) and slows the activities of anion channels (Suhita et al., 2004; Munemasa et al., 2007). These modulations are similar to those of ABA, suggesting an overlapping use of signaling components for closing of stomata. Constitutive expression of ERF1 activates the transcription of downstream effector genes, such as Plant Defensin1.2 (PDF1.2), to promote the ET response (Solano et al., 1998). JA and ET often induce the up-regulation of genes involved in plant defense, such as PDF1.2, VSP2, LOX2, and chitinases (Boter et al., 2004). Drought stress induces hypersensitive response mediated necrotic lesion in *Arabidopsis* plants (Tang et al., 2005). In *Arabidopsis*, the constitutive expression of pepper *CaHIR1* was reported to increase the resistance to *P. syringae* and *H. parasitica*, and the sensitivity to *Botrytis cinerea*, and it also conferred hypersensitivity to drought and salt stresses (Jung et al., 2008). Based on our results, we propose a model for the functional regulation of *Ca-AFP* under water-deficit conditions caused by water deficiency, mannitol, and PEG. The imposition of drought/osmotic stress causes hypersensitive response and production of stress related hormones like jasmonic acid, abscisic acid, and ethylene in plants (Haa et al., 2014). JA and ET trigger ERFs, which induce drought responsive genes (like *Ca-AFP*). These genes modulate the antioxidant activities, causing physiological changes and accumulation of osmoprotectants, resulting in water stress tolerance (Figure 9). *AtERF1* was reported to be highly induced under high salt and drought stress conditions and overexpression of *AtERF1* in *A. thaliana* resulted in enhanced tolerance to drought and salinity stress (Cheng et al., 2013). Involvement of JA trigger is indicated from the accumulation of transcripts of the jasmonic acid-marker gene, *AtVSP1*, after 15 days of drought stress in *A. thalaina* plants (Cho et al., 2013). In present study, we observed that the expression of *AtERF1* and *AtVSP1* was upregulated in transgenic *A. thaliana* plants under water-deficit stress conditions (Supplementary Figures S9A,B).

In conclusion, the overexpression of chickpea defensin gene in *A. thaliana* confers water stress tolerance. However, an understanding of the signaling pathways involved in the induction of *Ca-AFP* under water-deficit condition would need further investigation. Our results indicate that *Ca-AFP* can be a potential candidate gene for the development of drought tolerance trait in economically important crop plants.

## Author Contributions

MK (first author), PY, and SN conceived, designed and conducted the experiments. MK (last author) and MY analyzed the data and results. MK (first author), MY, and MK (last author) wrote the manuscript. MK (last author) monitored the experiments and critically commented on the manuscript. All the authors read and approved the final manuscript.

## Conflict of Interest Statement

The authors declare that the research was conducted in the absence of any commercial or financial relationships that could be construed as a potential conflict of interest.

## Footnotes

1 http:/www.ncbi.nlm.nih.gov/

2 https://www.ebi.ac.uk/Tools/msa/clustalo/

3 https://www.ncbi.nlm.nih.gov/gene/?term=LOC101512021
