# Supplementary material for: Overexpression of Chickpea Defensin Gene Confers Tolerance to Water-Deficit Stress in Arabidopsis thaliana
Source: Front Plant Sci. 2019 Mar 12;10:290. doi: 10.3389/fpls.2019.00290 (PMC6423178; doi:10.3389/fpls.2019.00290)
Supplement: Supplementary file 1 [file Data_Sheet_1.pdf]

Supplementary Material

Overexpression of Chickpea Defensin Gene Confers Tolerance to Water-Deficit Stress in *Arabidopsis thaliana*

Manoj Kumar, Mohd Aslam Yusuf, Pooja Yadav, Shiv Narayan, Manoj Kumar*
***Correspondence:** manojyd1234@gmail.com

Supplementary Figures

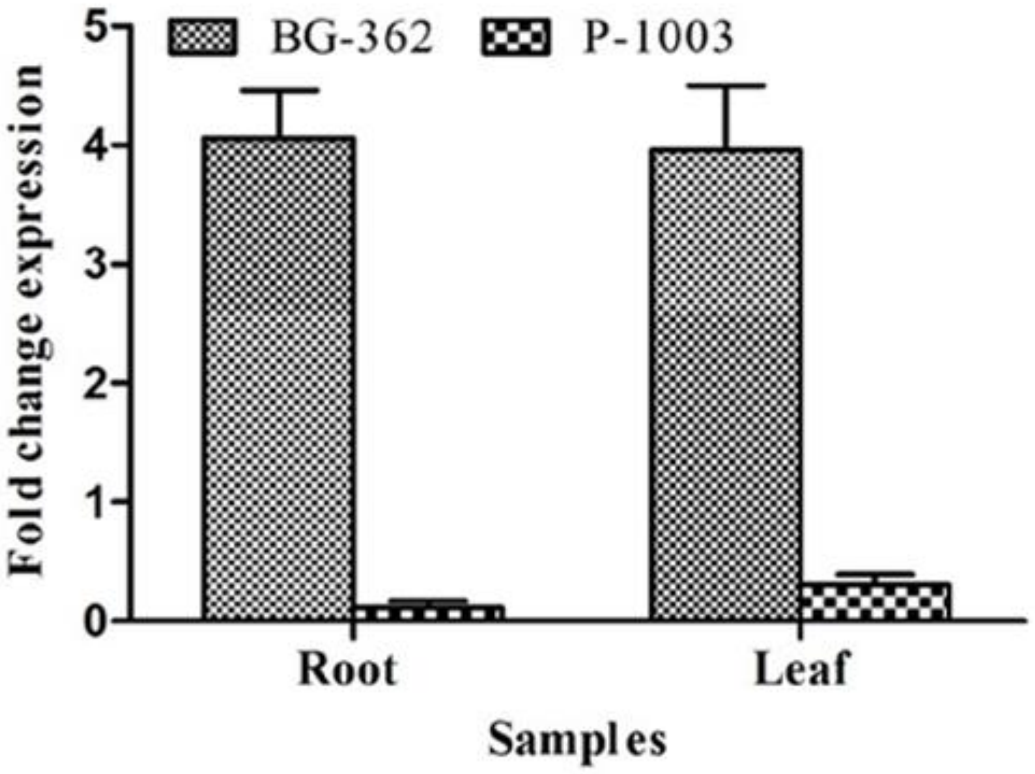

Supplementary Figure S1. Validation of *Ca-AFP* expression in chickpea root and leaf samples using qRT-PCR.

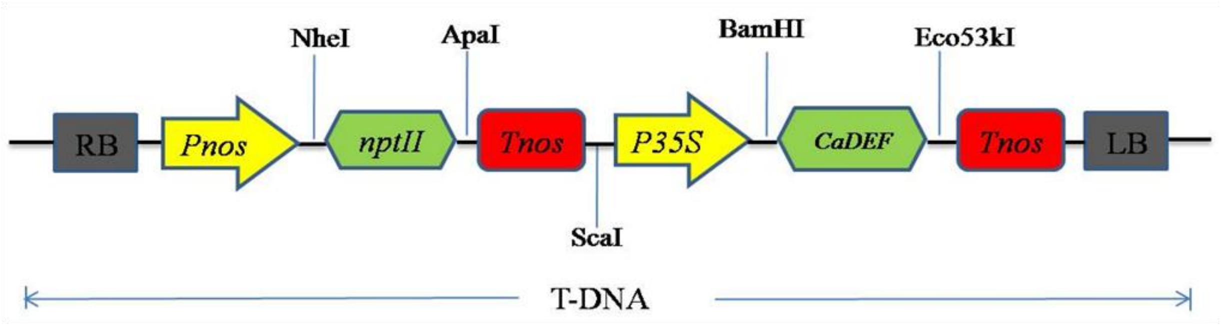

Supplementary Figure S2. *Ca-AFP* construct for plant expression in pBI121 vector for *Arabidopsis* transformation. *Pnos*-promoter sequence of the nopaline synthase, *Tnos*- terminator sequence of the nopaline synthase, *nptII*- neomycin phosphotransferase II, *P35S*- promoter sequence of cauliflower mosaic virus.

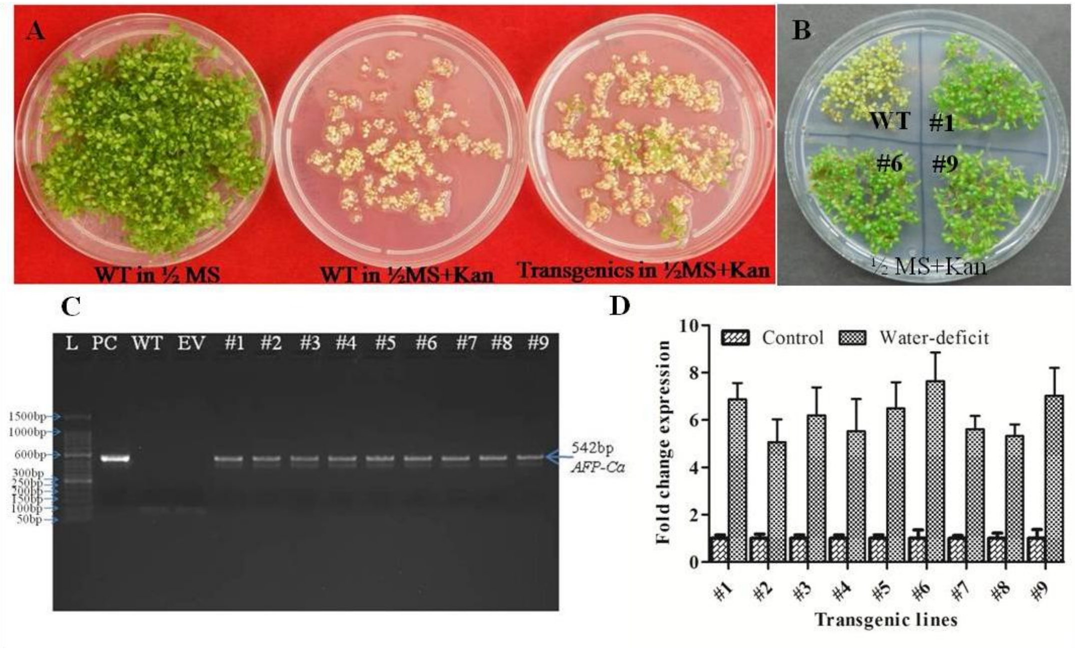

Supplementary Figure S3. Characterization of the integration and expression of *Ca-AFP* in transgenic *Arabidopsis* plants. (A) Wild-type (WT) and kanamycin (kan)-resistant T_1 transgenic *Arabidopsis* lines. Nine transgenic T_1 seeds were found to be kan-positive. **(B)** Homozygous T_3 transgenic lines, # 1, #6, #9, and WT plants growing on ½MS medium containing 50 mgL^{-1} kanamycin. **(C)** PCR amplification of *Ca-AFP* gene from the leaves of nine T_3 transgenic lines, WT, and empty vector (EV) transformed plants. L, 50-bp ladder; PC, positive control; WT, untransformed wild type; EV, empty vector; #1, #2, #3, #4, #5, #6, #7, #8, #9, transgenic samples. **(D)** Expression of *Ca-AFP* in the leaves of the nine T_3 transgenic lines (#1, #2, #3, #4, #5, #6, #7, #8, #9) as assessed using qRT-PCR under control and water-deficit stress condition. Values are means ± SE (n = 3).

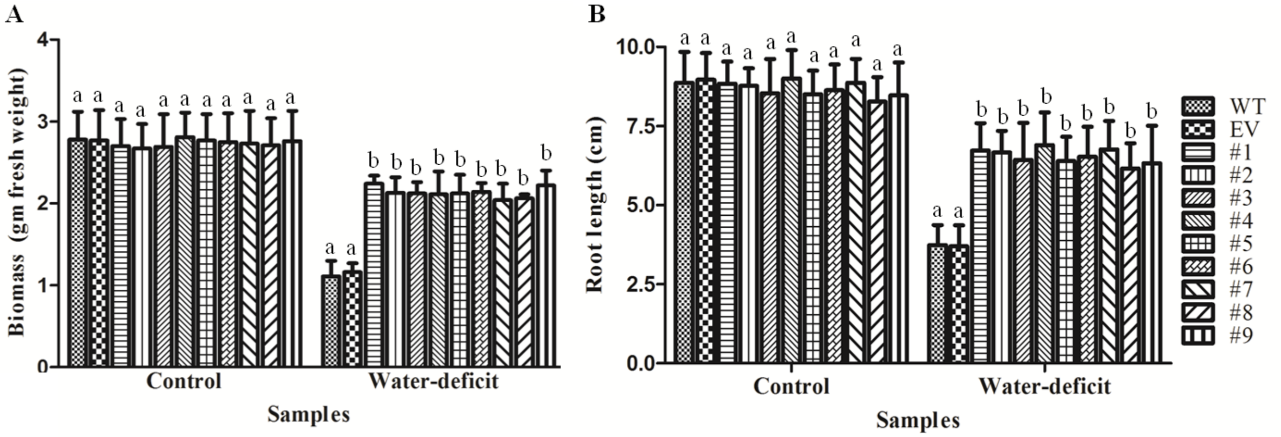

Supplementary Figure S4. Biomass and root length of different *Arabidopsis thaliana* lines under water-deficit stress condition. (A) Biomass and **(B)** Root length; of *Ca-AFP* (#1 to #9) and empty vector (EV) transformed line and wild-type (WT) plants under well-watered (control) conditions and after 15 days after of withholding water (water-deficit). Values are means ± SE (n = 3). Different letters above the bars indicate significant differences ($p < 0.05$) as analyzed by Duncan's Multiple Range Test.

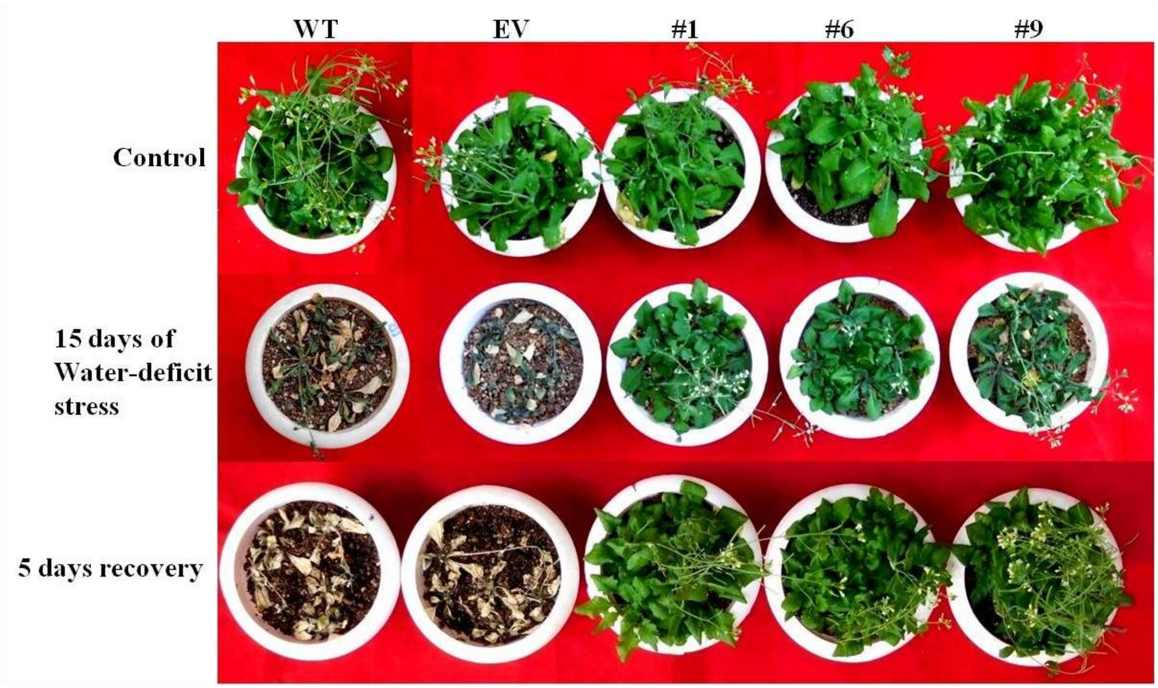

Supplementary Figure S5. Tolerance of different *Arabidopsis thalina* lines to water-deficit stress imposed at the pre-bolting stage. *Ca-AFP* (#1, #6, #9) and empty vector (EV) transformed lines and wild-type wild-type (WT) plants under control and water-deficit (for 15 days) conditions. Recovery of transgenic lines was determined after 5 days of re-watering.

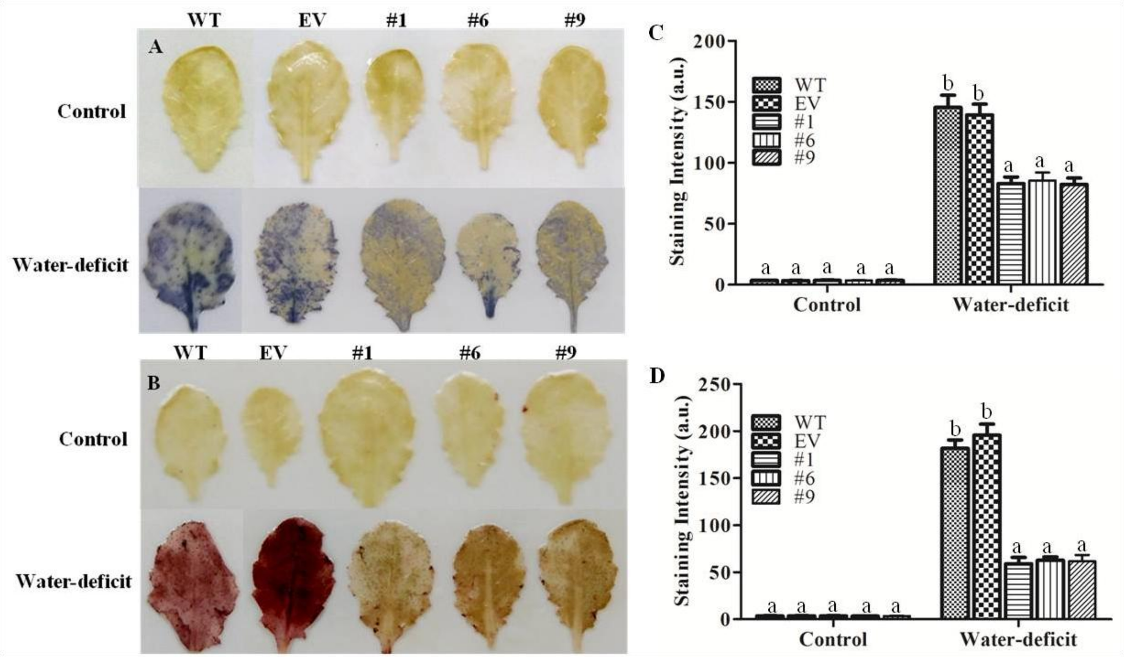

Supplementary Figure S6. Accumulation of reactive oxygen species under water-deficit stress in different *Arabidopsis thaliana* as assessed using histochemical staining. (A) Detection of O_2^- accumulation by nitroblue tetrazolium (NBT) staining. **(B)** Detection of H_2O_2 accumulation by di-aminobenzine (DAB) staining. For destaining and removal of chlorophyll, leaves were incubated in 96% ethanol, and were then photographed. WT: wild type plants; EV: Empty vector transformed plants; #1, #6, #9: transgenic lines expressing *Ca-AFP*. **(C)** Comparison of NBT staining intensity, and **(D)** Comparison of DAB staining intensity among WT, EV and transgenic lines (#1, #6, #9) under control and water-deficit condition. Values are means ± SE (n = 3). Different letters above the bars indicate significant differences ($p < 0.05$) as analyzed by Duncan's Multiple Range Test.

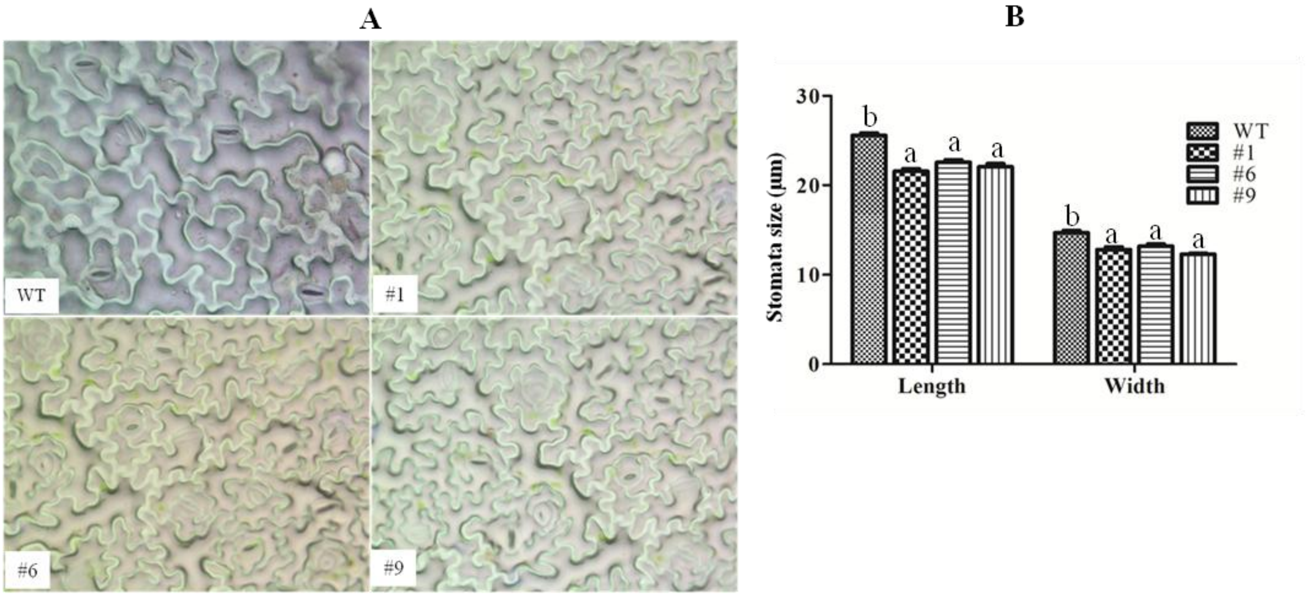

Supplementary Figure S7. Changes the stomata of wild-type (WT) and transgenic lines in response to water-deficit condition. **(A)** Morphology of stomata in the leaf epidermis of transgenic and WT plants photographed under Leica DM 2500 microscope at 40x magnification. **(B)** Comparison of the sizes of guard cells (width and length) was done under the microscope. Measurement of the stomatal size was performed using LAS V4.2 software. Five views from three replicate plants were observed microscopically for each of the transgenic and WT plant. Values are means of ± SE (n = 3). Different letters above the bars indicate significant differences ($p < 0.05$) as analyzed by Duncan's Multiple Range Test.

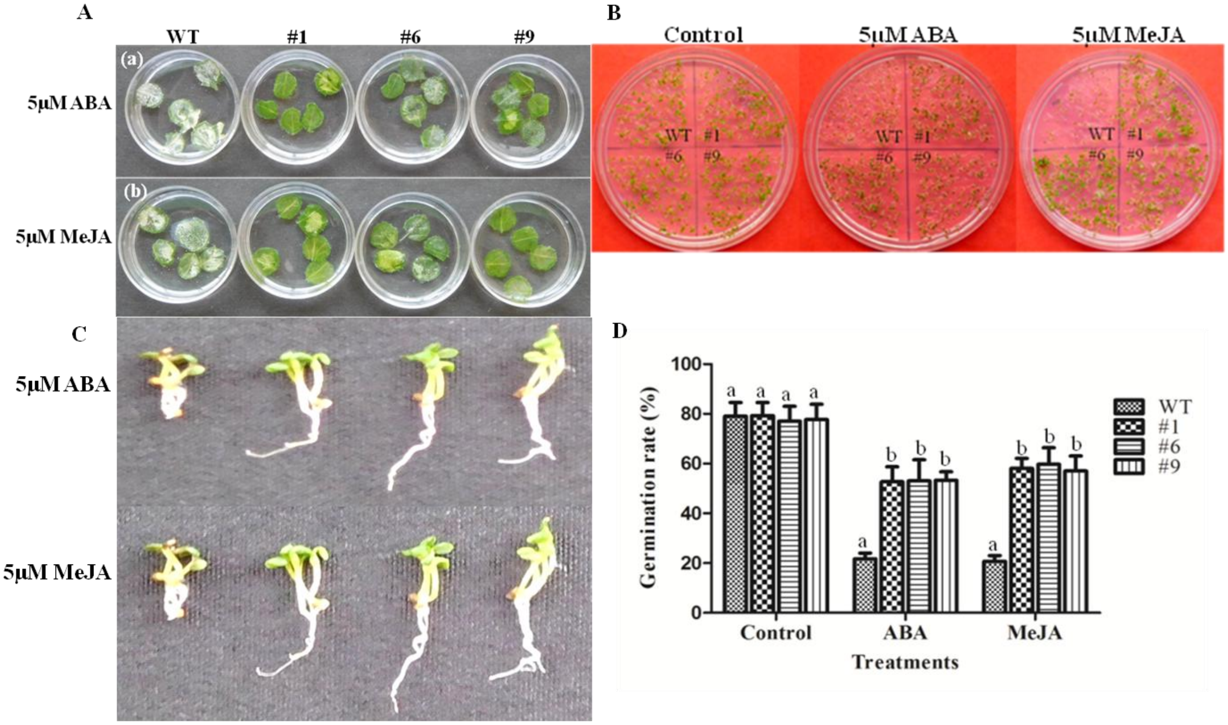

Supplementary Figure S8. Effect of abscisic acid (ABA) and methyl jasmonate on transgenic and WT plants. (A) Leaf discs of transgenic lines (#1, #6, #9) and WT plants were floated on **(a)** 5 µM ABA and **(b)** 5 µM MeJA overnight and photographed. **(B)** Germination of WT and transgenic seeds on ½ MS, 5 µM ABA, and 5 µM MeJA containing ½ MS medium. (**C)** Seedlings of WT and transgenic lines after 4 days of incubation in 5 µM ABA and 5 µM MeJA. (**D)** Histogram showing the germination rates of transgenic and WT seeds on ½ MS, 5 µM ABA, and 5 µM MeJA containing MS medium. Values are means ± SE (n = 3). Different letters above the bars indicate significant differences ($p < 0.05$) as analyzed by Duncan's Multiple Range Test.

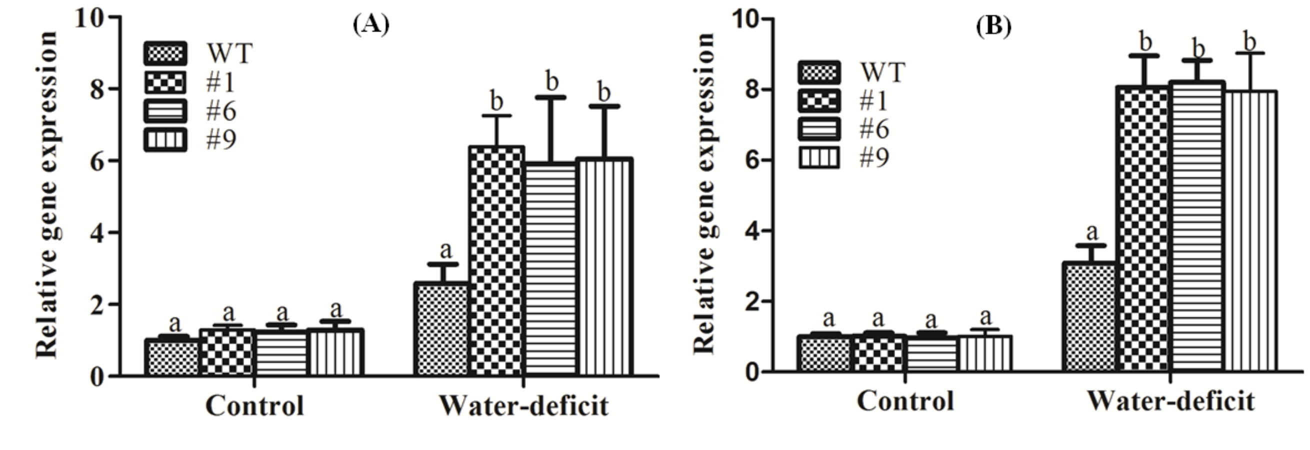

Supplementary Figure S9. Expression levels of ethylene and jasmonic acid related genes in transgenic lines and WT plants. (A) Expression of *ERF1* and **(B)** *VSP1*, measured using qRT-PCR in the wild-type (WT) and transgenic *Arabidopsis thaliana* (#1, #6, and #9) lines under control and water-deficit conditions. Values are means ± SE (n = 3). Different letters above the bars indicate significant differences ($p < 0.05$) as analyzed by Duncan's Multiple Range Test.
